# State-level policies alone are insufficient to meet the federal food waste reduction goal in the United States

**DOI:** 10.1038/s43016-024-01092-w

**Published:** 2025-01-09

**Authors:** Sarah Kakadellis, Selena Mao, Asch Harwood, Edward S. Spang

**Affiliations:** 1 Department of Food Science and Technology, University of California Davis, Davis, CA USA; 2 ReFED, Chicago, IL USA

**Keywords:** Environmental impact, Climate-change policy, Decision making

## Abstract

The United States Food Loss and Waste Reduction Goal seeks to reduce national food waste by 50%, down to 74 kg per capita, by 2030. Here we investigate state policies’ alignment with the federal goal across four policy categories. We develop a policy scoring matrix and apply it to wasted food solutions listed in the non-profit ReFED’s database to derive ranges of food waste diversion potential and projected generation across states. On the basis of state policies alone, no state can meet the federal target. We estimated a diversion potential of 5–14 kg per capita and a food waste generation of 149 kg per capita nationally in 2022, equivalent to the 2016 baseline. Without additional intervention at the state and federal level promoting a shift from food waste recycling towards prevention, rescue and repurposing, food generation in the United States will probably remain high.

## Abstract (web summary)

A modelling study shows that the emphasis on recycling food waste over prevention, rescue and redirection impedes the progress of the United States Food Loss and Waste Reduction Goal to halve food waste by 2030.

## Main

Producing uneaten food squanders limited resources and poses substantial environmental, economic and social burden on already strained food systems. Globally, 13% of food produced is lost at early stages of the food supply chain between harvest and distribution, referred to as food loss^1^. An additional 19% of global food production is attributed to food waste occurring at consumer-facing stages, defined as food leaving the human food supply chain from retail, food service and residential sectors^1^. Food systems worldwide contribute a third of anthropogenic greenhouse gas (GHG) emissions^2^. Food waste alone has been estimated to account for 8–10% of those emissions globally^1^ and 58% of methane emissions from municipal landfills in the United States^3^. Preventing unnecessary food loss and waste (FLW) remains a challenge and underscores the complexity and inefficiency of contemporary food systems. Food systems are sprawling decentralized systems composed of a multitude of stakeholders, including producers, manufacturers, distributors, retailers, food service businesses and consumers. Causes of FLW are diverse and vary by food type, sector and stage along the food supply chain^4^, which means a portfolio of solutions is required.

Recognizing the importance of FLW reduction, the United Nations introduced a target to halve per capita global food waste and reduce food loss by 2030 as part of its Sustainable Development Goals (SDGs) framework^5^. SDG 12.3 sits under the broader goal of SDG 12 to promote sustainable consumption and production patterns^5^. By 2022, national governments and regional blocks representing 55% of the global population had set specific targets in line with SDG 12.3^6^, including the United States—the third largest FLW generator worldwide after China and India^7^.

To align with SDG 12.3, the US Department of Agriculture (USDA) and the US Environmental Protection Agency (EPA) set a national goal to halve the 107 kg per capita of food waste landfilled or incinerated in 2016 by 2030 (ref. ^8^). In 2021, the EPA revised its targetable baseline, informed by a new definition of food waste, to emphasize minimizing unnecessary GHG emissions^8^. The EPA redefined food waste as ‘any food [from retail, food service and residential sectors] leaving the human food supply chain’^8^, referred to here as EPA-2021. This includes any food going to landfill and incineration, as in the 2016 baseline (EPA-2016), and sewer and biological food waste recycling strategies originally included as relevant diversion pathways. In contrast, the term wasted food encompasses food waste and food re-entering the food supply chain, for example, donated surplus food and edible food fractions used as animal feed or repurposed to produce other food products. This reinterpretation raised the applicable amount of food waste to be halved to 149 kg per capita and the reduction target to 74 kg per capita.

While ambitious, the US FLW reduction goal primarily serves as federal guidance for states and municipalities, which are responsible for solid waste management^9^. Existing federal policies relevant to FLW are limited in scope and include both voluntary and regulatory instruments aimed at incentivizing food donation (from a food safety/information perspective), regulating use-by date labelling (currently for infant formula only) and determining which food streams can be redirected to animals^9^.

Achieving the federal goal of halving per capita food waste by 2030 will require a range of minimization/management policies implemented by states and municipalities. These policies can be categorized into the following themes: (1) prevention (narrowing resource loops, for example, reducing plate waste), rescue (slowing resource loops), further divided into (2) direct rescue (for example, food donation) and (3) indirect rescue, or repurposing (for example, animal feed), and (4) recycling (closing resource loops, for example, composting)^9,10^. Historically, policies on food waste have focused on recycling as a means of managing waste to safeguard public health and, more recently, motivated by environmental concerns. Hence, there has been limited attention to enabling prevention, rescue and repurposing^11^. Additionally, policies rarely account for interdependencies and potential conflicts between them, including competing priorities between rescuing food surplus and valorizing food waste^12^. This has prompted criticism of recycling narratives framing food waste predominantly as a business opportunity and entrepreneurial asset^13^, potentially normalizing or even encouraging food waste generation.

Despite ambitious federal goals, there is limited knowledge on whether existing state and local policies will enable the United States to meet its 2030 reduction target. Sparse, decentralized data collection, discrepancies in quantification methods and reporting practices and limited collaboration efforts represent major barriers to quantify policy effectiveness^9^. To address this gap, we propose a method to quantitatively estimate the food waste diversion potential of policies and project food waste generation levels among states. By quantitatively assessing the amount of food waste diverted from destinations in line with EPA-2016 and EPA-2021 definitions, this study seeks to answer the following research questions:

1. Which states, given their existing policies, are on a track to reach the federal goal of halving per capita food waste by 2030?
2. Do policies enabling prevention, rescue, repurposing and recycling strategies differ in their relative contribution across states?
3. Informed by questions (1) and (2), how does this variance impact the federal goal?

To address these questions, we estimated the food waste diversion potential for each state across four broad policy categories: prevention (date labelling), rescue (liability protection and tax incentives), repurposing (animal feed) and recycling (organic waste bans and waste recycling laws). Diversion potential predictions leveraged publicly available data on food waste diversion strategies from the US non-profit ReFED, which specializes in data-driven research to reduce FLW. By estimating the maximum and likely amount of food waste that could be diverted through policy measures, this study offers unique insights into the effectiveness of existing food waste diversion policies. We show that while numerous states have implemented policies aimed at minimizing and/or redirecting food waste, the United States is unlikely to meet its 2030 goal, based on state policies alone. We argue that state policies designed to support the goal of reducing food waste are either non-existent or not robust enough and that existing policies overemphasize recycling strategies.

## Results

### Food waste diversion progress at the federal level

**Fig. 1:**
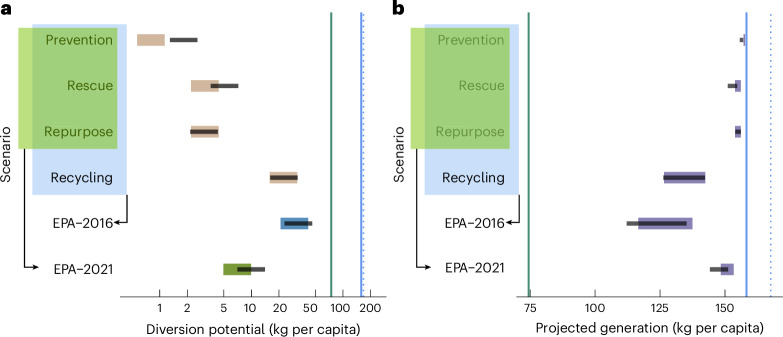
Likely food waste diversion potential and projected food waste generation at the federal level. Federal food waste diversion/generation goal (green) and weighted (blue, solid fill) and non-weighted (blue, dotted) food waste generation average across 50 states in 2022. **a**,**b**, Coloured segments represent the ranges of diversion potential (**a**) or generation (**b**) for the baseline scenario. Additional solutions modelled in the alternative scenario are depicted as black lines. The EPA-2016 scenario represents the sum (Σ) of diversion potentials from all policy categories, whereas the EPA-2021 scenario reflects the updated EPA definition, which excludes recycling. Projected food waste generation ranges (**b**) were obtained by extracting food waste diversion potential ranges from current food waste generation levels (2022). Federal averages across each category/EPA scenario ranges represent weighted averages based on state population. The *x* axis in **a** is on a log2 scale. Source data

We investigated the contribution (weighted across the 50 states) of each policy category towards overall food waste diversion potential at the federal level (Fig. 1a). The recycling policy category represented the single largest diversion potential. Despite this substantial contribution, in the most optimistic case under the EPA-2016 scenario (in blue), the likely food waste diversion rate fell short of the federal target (46 vs 74 kg per capita). In the updated EPA-2021 scenario (in green), which excludes recycling, the most optimistic diversion rate reached 14 kg per capita. This corresponds to 18.92% of the diversion target, or a 9.46% reduction in absolute terms from the 2016 baseline of 149 kg per capita. We then subtracted ranges of likely food waste diversion potential from 2022 food waste generation to derive food waste generation projections (Fig. 1b) and assess progress towards the 2030 goal. Under the current EPA definition (EPA-2021), the projected food waste generation averaged 149 kg per capita (125 kg per capita under EPA-2016), double the federal target and, notably, identical to the 2016 baseline.

### Food waste diversion progress at the state level

**Fig. 2:**
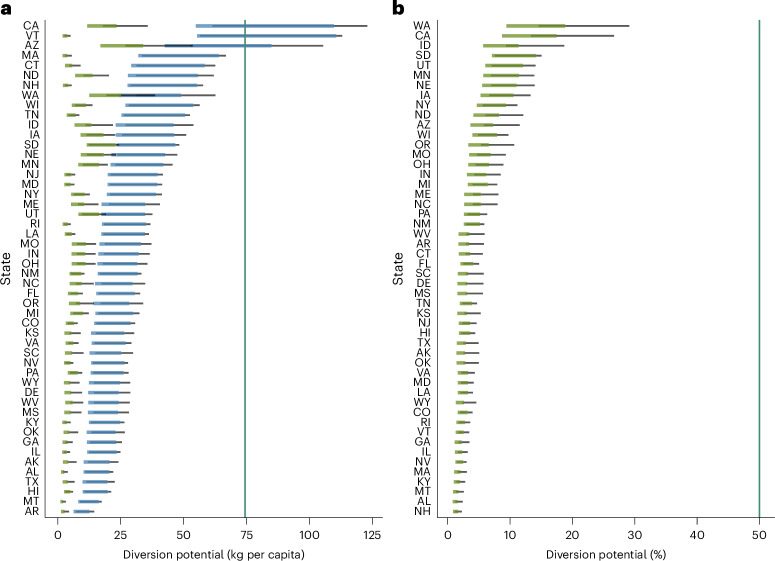
Absolute and relative likely food waste diversion potential across US states. **a**,**b**, Absolute (**a**) and relative (**b**) likely food waste diversion potential across US states in 2022. Federal food waste diversion target is shown in green. Coloured segments represent likely ranges for the baseline scenario. Additional solutions modelled in the alternative scenario are depicted as black lines. Blue segments represent diversion pathways as initially defined by the EPA (EPA-2016), whereas green segments reflect the updated EPA methodology (EPA-2021), which excludes recycling. The *x* axis in **a** is on a log10 scale. Relative contributions in **b** were derived from 2022 state food waste generation (Fig. 3) and plotted for the EPA-2021 scenario only. Source data

**Extended Data Fig. 1:**
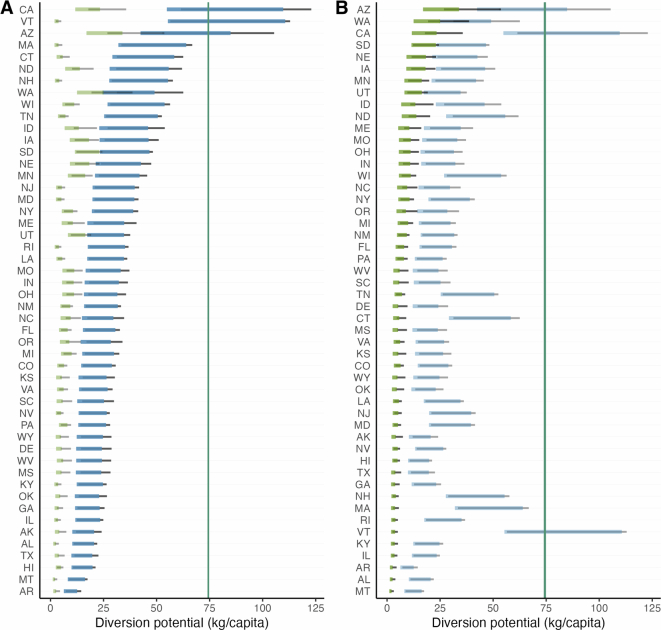
Likely food waste diversion potential across US states (2022) arranged hierarchically based on the EPA’s original (A) and revised (B) food waste definition. The green line corresponds to the federal food waste reduction goal (74 kg per capita) by 2030. Coloured segments represent the likely range for the baseline scenario, while additional solutions modelled in the alternative scenario are depicted as black lines. Blue segments represent diversion pathways as originally defined by the EPA (EPA-2016), while green segments reflect the updated definition (EPA-2016), which excludes recycling. The left panel is identical to Fig. 2a, with additional shading to facilitate visualization and comparison.

We then modelled likely ranges of food waste diversion potential under both EPA-2016 and EPA-2021 scenarios across states (Fig. 2a). When all policy categories were considered (EPA-2016, in blue), results showed a tenfold difference between the bottom (Arkansas) and top (California) performers, with a uniform spread over this range. Even under the most ambitious EPA-2016 scenario, only California, Vermont and Arizona were projected to achieve a 74 kg per capita diversion rate (green line). Notably, under the EPA-2021 scenario (in green), all 50 states fell short of the 2030 diversion target. States were unevenly affected by this revision (Extended Data Fig. 1 and Extended Data Table 1).

**Fig. 3:**
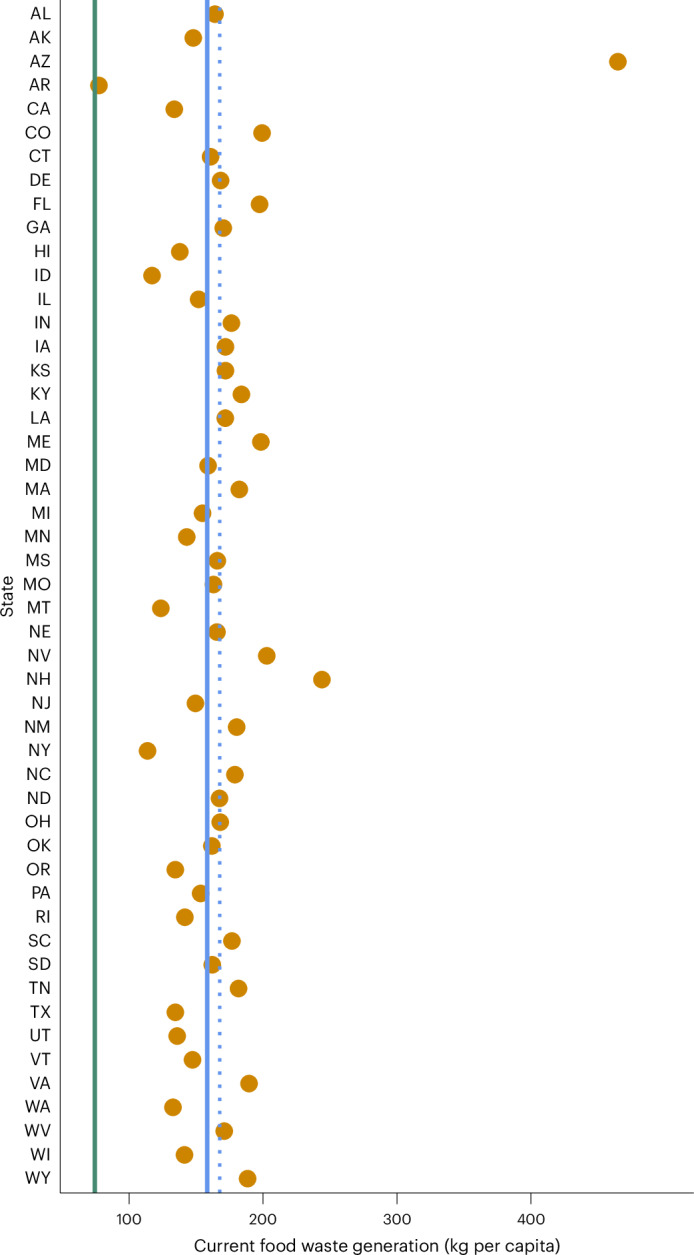
Current per capita food waste generation levels across US states. Data represent food waste as defined by the EPA post 2021. Green vertical line shows food waste generation goal (74 kg per capita); blue vertical line (solid fill) shows current (2022) weighted food waste generation average across 50 states (158 kg per capita), relative to individual states’ populations and dotted blue vertical line shows current (2022) non-weighted food waste generation average across 50 states (168 kg per capita), following a scaling-up approach as adopted by the EPA. Source data

While quantifying ranges of likely food waste diversion provides an insight into the absolute contribution of state policies towards the 2030 goal, assessing the alignment of individual states with the federal target requires consideration of those contributions relative to states’ current food waste generation levels (Figs. 2b and 3 and Extended Data Table 2). Most states displayed levels within 0.5–1.5 × the current federal average (79–237 kg per capita), except for Arkansas (78 kg per capita), Arizona (465 kg per capita) and New Hampshire (244 kg per capita) (Fig. 3).

Across all scenarios, states were estimated to divert 4.99% of their respective food waste generation. In the most ambitious scenario, Washington was estimated to be able to divert close to a third (29.12%) of its current food waste generation, closely followed by California (26.68%).

**Fig. 4:**
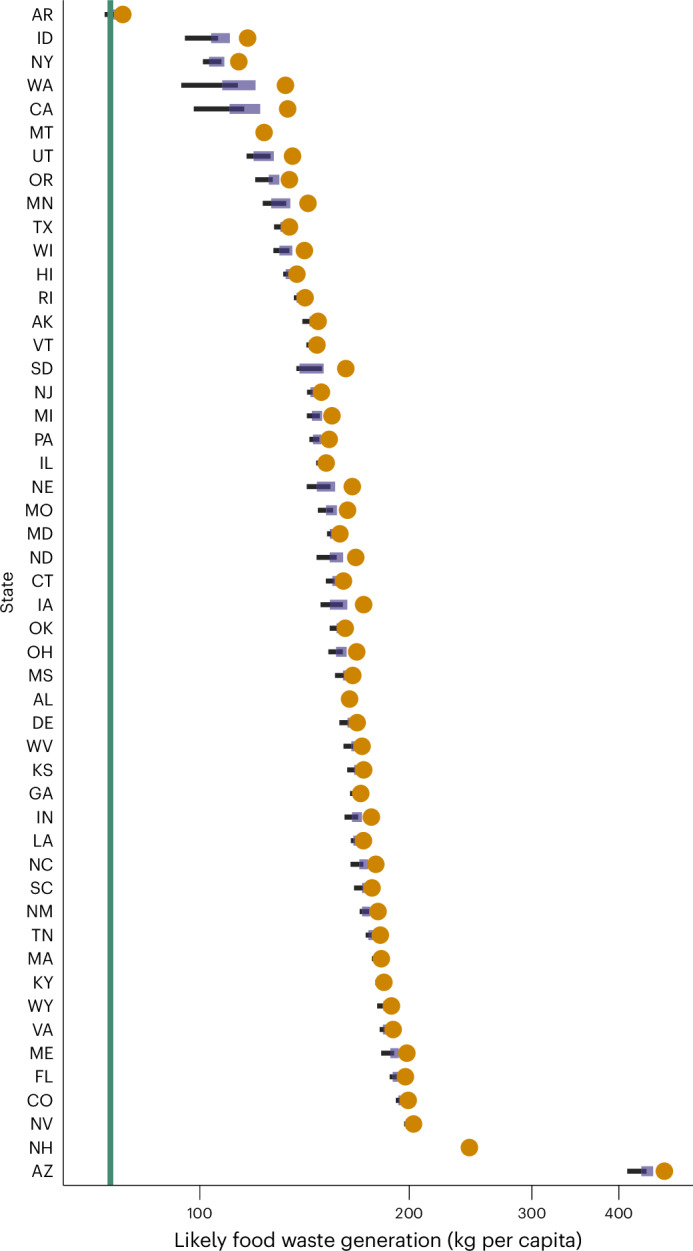
Likely food waste generation levels across US states under the EPA-2021 scenario. In the EPA-2021 scenario, the contribution of recycling policies is excluded to reflect the EPA’s updated food waste definition. The green line shows federal food waste generation target and orange circles show state per capita food waste generation in 2022. Coloured segments represent the likely ranges for the baseline scenario, whereas additional solutions modelled in the alternative scenario are depicted as black lines. The *x* axis is on a log10 scale. Source data

These relative contributions helped derive projected food waste generation levels (Fig. 4 and Extended Data Table 3). Despite a limited diversion potential (both in relative and absolute terms), based on current state food waste generation levels, Arkansas emerged as the only state capable of achieving 74 kg food waste per capita, though only in the best-case scenario.

### Contribution of policy categories towards diversion potential

**Fig. 5:**
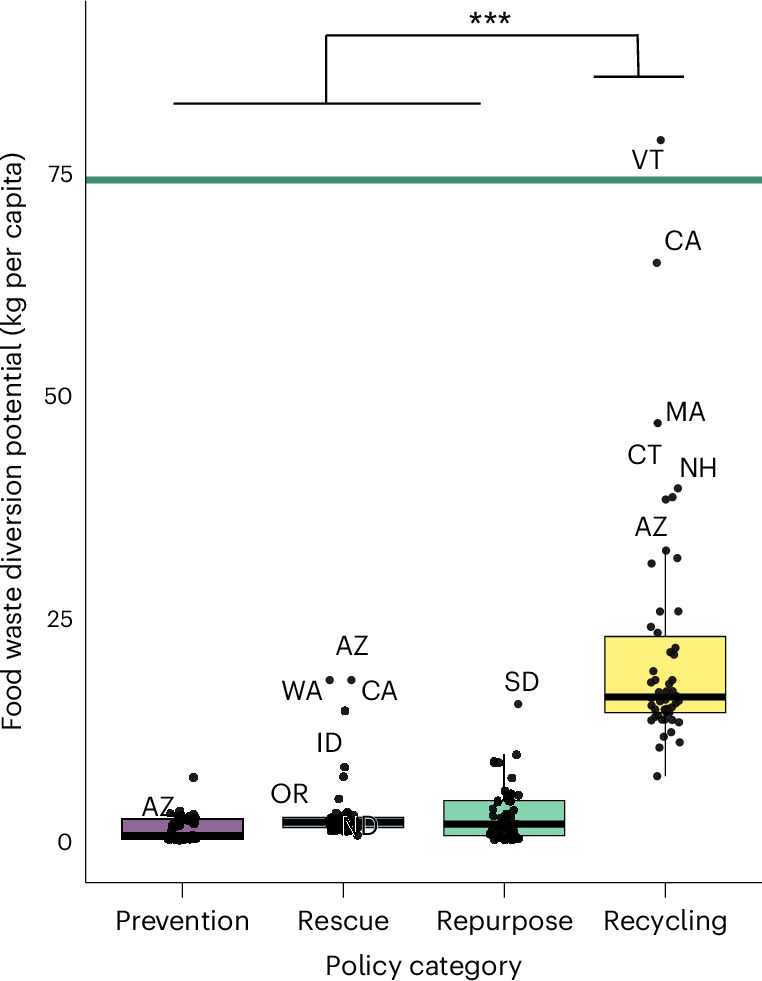
Contribution of individual policy categories towards likely food waste diversion potential. Diversion potential values are based on the EPA’s original methodology (EPA-2016 scenario), which includes recycling. For each box plot, the centre line shows the median, the box limits show the 25th and 75th percentiles and the whiskers extend to the minimum and maximum, defined as the 25th and 75th percentiles ± 1.5× the interquartile range, respectively. Each data point corresponds to the state average based on four data points (that is, high and low conversion factors for both baseline and alternative scenarios), with *n* (per policy category) = 50. Only outliers (above and below the maximum and minimum, respectively) were labelled to avoid cluttering. The green line reflects the federal food diversion goal. One-way analysis of variance (ANOVA) with post-hoc Tukey’s honest significance test: *F*(3, 196) = 85.71 (*P* < 0.001 (***)). Additional statistical analysis was also conducted separately for the baseline and alternative scenarios, yielding similar results (Extended Data Fig. 2). Source data

**Extended Data Fig. 2:**
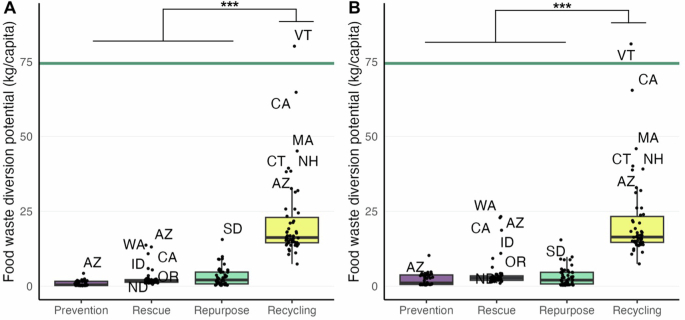
Contribution of individual policy categories toward likely food waste diversion potential (2022) for baseline (A) and alternative (B) scenarios. Diversion potential values are based on the EPA’s original methodology (EPA-2016 scenario) to be able to compare recycling with the remaining policy categories. For each box plot, the centre line shows the median, the box limits show the 25th and 75th percentiles, and the whiskers extend to the minimum and maximum, defined as the 25th and 75th percentile -/+ 1.5× the interquartile range (IQR), respectively. Each data point corresponds to the state average based on four data points (that is high and low conversion factors for both baseline and alternative scenarios), with n (per policy category) = 50. Only outliers (above and below the maximum and minimum, respectively) were labelled to avoid cluttering. The green line reflects the federal food diversion goal. One-way analysis of variance (ANOVA) with post-hoc Tukey’s honest significance test: (A) F(3, 196) = 93.709 (p < 0.001 (***)) and (B) F(3, 196) = 77.715 (p < 0.001 (***)).

The overall left shift in food waste diversion potential in Fig. 2a highlighted the dominance of recycling strategies in the policy landscape, which we further investigated in Fig. 5 and Extended Data Fig. 2. We found a statistically significant difference in diversion potential ranges across policy categories (one-way ANOVA: *F*(3, 196) = 85.71, *P* < 0.001). Assessing diversion potential by policy category yielded additional insights into the disproportionate impact of the revised EPA definition on individual states, which we discuss next.

## Discussion

The likely food waste diversion potential was modelled for each US state under three modelling parameters to account for: (1) additional solutions reflecting the indirect impact of behavioural campaigns on food waste diversion across the supply chain (baseline vs alternative scenarios, corresponding to coloured segments and black lines, respectively); (2) policy effectiveness (low and high factor ranges, corresponding to the lower and upper limit of diversion potential, respectively) and (3) changes in federal definition and methodology for food waste quantification (EPA-2016 and EPA-2021 scenarios), yielding four diversion potential ranges for each state. On the basis of current state policies and despite noticeable differences between individual states, none of the 50 states included in the analysis are likely to meet the revised federal goal of halving per capita food waste by 2030, except Arkansas in the most optimistic scenario only (which includes additional solutions on top of the baseline scenario and considers a high policy implementation score). Arkansas’ unusually low current food waste generation represents an important caveat and should be interpreted with caution whereas more accurate food waste characterization data become available. Nonetheless, these projections are alarming, given that the target is 6 years away from when this analysis was conducted and given that 2022 data showed that food waste generation has increased overall. They also bring to light three intersecting questions/implications when thinking about effective policy design: (1) *why* are state policies failing to meet goal(s) set by the federal government? (2) *how* (from a policy perspective) do the best and worst performing states differ? Lastly, (3) *what* does an ideal food waste diversion policy look like?

Comparing ranges of likely food waste diversion potential between EPA-2016 and EPA-2021 scenarios provided some insights into why states fall short of the 2030 target. The significant and disproportionate contribution of recycling strategies among state policies highlights a misalignment between state and federal priorities. This observation aligns with scholarly literature pointing at the dominance of a recycling-oriented narrative in the policy landscape^9,11,13^. This is expected, given that policies prioritizing food waste recycling strategies are perceived as compatible with current economic paradigms without confronting the status quo and questioning current production and consumption patterns^11^. Nonetheless, the emphasis on recycling over the prevention, rescue and redirection of wasted food (towards upcycled food products and animal feed) was not reflected uniformly in our analysis, as evidenced by the uneven gap between EPA-2016 and EPA-2021 scenarios across states (Fig. 2a).

Of the nine states that have enacted organic waste bans and/or waste recycling laws, six scored considerably worse once recycling solutions were excluded from the model (Extended Data Table 1), suggesting that strong recycling policies do not guarantee parallel legislation on food waste prevention, rescue and repurposing and may even come into competition between targets^12^. Vermont, the first state ever to introduce a ban on the disposal of food scraps in residual waste or landfills in 2012^14^ and second-highest ranking in the original scenario, epitomizes this tension, scoring 45th when excluding food waste recycling policies from the model. This also echoes scholarly literature on food waste-related legislation in the European Union. Indeed, despite the introduction of mandated separate organic waste collections from business and households, the amended EU Waste Framework Directive and Landfill Directive have both failed to set an explicit food waste target and rely predominantly on soft instruments for food waste prevention, limiting accountability and the effectiveness of strong preventative measures^15–17^. Nonetheless, a legally binding target of 30% per capita food waste reduction was proposed by the European Commission as part of its commitment to the European Green Deal and in line with SDG 12.3^18^.

In contrast to Vermont, the consistently high ranking of California and Washington reflects a more comprehensive approach by those states to address wasted food, encompassing both food surplus prevention and distribution and waste recycling. Acknowledging the multi-faceted impact of food waste on food insecurity, global warming and climate resilience, California’s 2016 Short-Lived Climate Pollutant Reduction Strategy (SB 1383) set reduction targets for the landfilling of food waste, introduced mandatory separate organic waste collection from all residents and businesses and required certain food service businesses to donate 20% of currently disposed surplus food^19^. California also offers some of the most robust liability protection regulations and tax incentives for food donations^20^.

In some cases, prioritizing one policy category over another may be best understood in the relevant geographical and economic context. South Dakota and Nebraska both achieved high food waste diversion potential rankings under the EPA-2021 scenario, and Fig. 5 pointed at South Dakota’s leading position on animal feed policies. South Dakota generally does not have any restriction for feeding food waste to animals. Nebraska requires animal-derived food streams to be treated according to the Commercial Feed Act (if fed to animals) but does not restrict its use as animal feed^21^. The United States has the largest fed-cattle and poultry industries in the world^22^, with both South Dakota and Nebraska among the top five biggest cattle industries among US states, each accounting for over 5% of total cattle raised nationally^23^. A favourable legislative and regulatory policy landscape for the use of wasted food for animal feed probably reflects the importance of the animal agricultural sector in those states.

The performance of individual states also needs to be considered in relative terms, as illustrated in Figs. 2b and 4. Arizona scored highly across all scenarios when considering food waste diversion, in part due to its substantial diversion potential through food rescue. However, the state also generated significantly more food waste than any other state on a per capita basis (465 kg per capita, nearly thrice the national 2022 weighted average). Similarly, Arkansas performed poorly across both EPA-2016 and EPA-2021 scenarios from a food waste diversion perspective. However, its per capita food waste generation is the lowest in the country (78 kg per capita), less than half the national average (Fig. 3). It is thus closer than any other state to reaching the federal target (< 1 kg short on average under the EPA-2021 definition). Thus, whereas Arizona’s food rescue policy landscape, which includes both food liability protection and tax incentives, was considered moderate overall in the model calculations, the absolute volume of food surplus inflated its food waste diversion potential.

The disparity in policy rating and predicted effectiveness across states calls for a scholarly discussion on how an optimal policy or policies would be formulated (if such an ideal may even exist) within a certain social, spatial and temporal context. Given that halving global FLW could result in a 24% reduction in GHG emissions from food systems between 2020 and 2100 relative to business-as-usual scenarios^24^, with trickle-down effects on climate and social resilience, ensuring effective policy design in the following years represents a vital component of a just and sustainable transition. Today, food systems are characterized by complex supply chains with a diverse range of stakeholders with contrasting, and at times competing, priorities^25^. Implementing ambitious and effective policies that are also deemed acceptable across stakeholders will remain a challenge and requires strong political will to change. The design of potential strategies needs to be carefully examined, as the effectiveness of similar measures is perceived differently depending on the implementation strategy^25^. This highlights the importance of distinguishing between implementing policy and achieving the corresponding policy goals. For instance, the independent oversight agency Little Hoover Commission released a report recommending a temporary hold on California’s SB 1383 to allow the legislature to reassess its implementation and invest more heavily in education campaigns, after the state missed its 2020 target on the landfilling of food waste^26^.

Furthermore, consistent messages with a clearly defined purpose and well-aligned implementation strategies are essential to policy narratives^27^. Indeed, how policies are framed matters. Public support for more aggressive food waste reduction targets increased when the rationale for tighter regulation emphasized social norms^28^. Policies also need to consider the local context into which they are ultimately implemented^27^. This requires distinguishing between the *character* (that is, the characteristics of a given policy instrument, for example, cost, intrusiveness, precision of targeting and so on) and the *context* (social, temporal, economic and so on) of policy design to effectively match the theoretical potential of policy tools with the reality of their local context^29^.

This study presents some modelling limitations pertaining to (1) the solutions modelled, (2) policy scoring, (3) the scope of policies included and (4) the reliability and validity of food waste estimates. First, only 14 out of 42 solutions currently modelled in ReFED’s Solutions Database were included in the analysis based on their relevance to the policy intervention types assessed. Even when implementing all 42 modelled solutions (without accounting for policy scoring), it was estimated that only a 23% net reduction in surplus food could be achieved^30^. With an additional 31 potential yet currently unmodelled solutions, more research and partnerships are needed to understand and model the contribution of those unmodelled solutions towards food waste diversion. In addition, to prevent double counting, most solutions were either assigned to a single policy category or split equally across two policy categories. This approach led to quantifying a total diversion potential range by summing the food waste diversion potential of individual policies through additive uncertainty. However, policies often exist alongside other policies with multiplicative uncertainty^31,32^, which may be considered in future models.

Second, the policy scoring matrix, which translated qualitative policy assessment into quantitative ranges, relied on expert-derived approximation. Further research is needed to develop a more granular and nuanced policy scoring system (for example, evaluating the strength of date labelling policy based on a weighted average across individual food types and their respective representation in food waste streams) and additional metrics/policy dimensions. Initial efforts dedicated to developing an institutional grammar to analyse the syntactic structure of policy documents represents an important step towards the systematic evaluation of policies through computational text analysis and natural language processing^33^.

Third, our model only accounted for policies implemented by state (and, to a limited extent, federal) governments. Actions taken by the private sector may play a non-trivial contribution towards meeting the 2030 goal, alongside a more accurate assessment of federal policies. For example, the Pacific Coast Food Waste Commitment, launched in 2019, represents one of the largest public–private partnerships dedicated to implement measurable actions to halve wasted food by 2030 in the Pacific Coast region^34^. By 2023, unsold food rates among retail signatories had decreased by 28% (ref. ^34^).

Lastly, it is worth noting that our model ultimately relies on the reliability and validity of the underlying database on food waste estimates. ReFED’s Insights Engine Food Waste Monitor adopts a top-down, mass-balance approach starting with broad estimates of food supply entering each food sector, after which fixed surplus, cause and destination rates are applied. Improving the accuracy and granularity of the data would involve using waste characterization studies and data reported by waste collected, which remain limited and highly dependent on the completeness of individual reporting sources.

In conclusion, our analysis represents a stepping-stone towards quantitative assessment of policy effectiveness in the wasted food landscape. Though we found that state policies alone are insufficient to meet the federal target and have had limited success at curbing per capita food waste generation over time, they represent important safeguards to prevent exacerbating this issue further. Without a counterfactual, their efficacy at addressing food waste reduction remains to be determined and deserves further attention. Nonetheless, the current prioritization of food waste recycling policies calls for a more holistic reframing of wasted food policies to anchor them within a whole-systems approach. This would mean capturing (and later mitigating) the impacts of wasted food throughout its life cycle to build truly sustainable and resilient food systems.

## Methods

### Overall conceptual framework

To investigate policy effectiveness on food waste diversion across the United States, the following methodological steps were conducted: (1) quantified current food waste generation levels (as per the EPA-2021 definition) to establish the *maximum* food waste diversion potential at the state and federal level; (2) identified relevant food waste diversion solutions and assigned them to one (or two) policy intervention types (date labelling, liability protection, tax incentives, animal feed and organic waste bans/waste recycling laws) and corresponding policy category; (3) calculated the *applicable* food waste diversion potential for each policy category and state based on the solutions identified and attributed causes of food waste arising; (4) developed a conversion matrix to translate qualitative policy assessment scores into a quantitative range; (5) applied the conversion matrix to the applicable food waste diversion potential to estimate the *likely* range of food waste diversion achievable based on current policies; (6) revised the likely range of food waste diversion potential in line with the EPA’s updated definition of food waste diversion and (7) estimated the projected food waste generation and evaluated the role played by the dominant narrative of food waste management.

All datasets were extracted as comma-separated values (CSV) files and imported into R (v4.4.1) via RStudio (v2024.04.2 + 764). The pipeline for data extraction, analysis and visualization and input and output CSV files are publicly available on the platform GitHub (github.com/s-kakad/wasted_policy).

### Total food waste generation quantification

Total food waste generation (in million US short tons (Mt)), which represents the maximum amount of food waste and surplus to be targeted, was obtained for 2022 from ReFED’s Insights Engine Food Waste Monitor (https://insights-engine.refed.org/food-waste-monitor). The Food Waste Monitor is a centralized repository based on data from over 80 public and proprietary databases and provides estimates of food loss, food waste and food surplus, broken down by sector (farm, manufacturing, food service, retail and residential); food types (for example, produce, breads and bakery, dairy, eggs and so on) and their causes (for example, excess, food safety concerns, by-products from processing and so on); and destinations (for example, food donation, animal feed, composting, landfill and so on) for each state. To align with the EPA’s definition and methodology, which aims to halve food waste from retail, food service and residential sectors, the farm and manufacturing sectors were omitted when quantifying total food waste generation. Per capita food waste generation (expressed as kg per capita) was calculated at the state level based on 2022 data obtained from census data:

1 $$\begin{array}{l}{\mathrm{State}}\; {\mathrm{per}}\; {\mathrm{capita}}\; {\mathrm{wasted}}\; {\mathrm{food}}\; {\mathrm{generation}}\\=\frac{{\mathrm{Total}}\; {\mathrm{food}}\; {\mathrm{waste}}\; {\mathrm{generation}}\; {\mathrm{of}}\; {\mathrm{state}}\;\left({\mathrm{Mt}}\right)\times 907.185\;({\mathrm{kg}}\;{\mathrm{Mt}}^{-1})}{{\mathrm{Population}}\; {\mathrm{size}}\; {\mathrm{of}}\; {\mathrm{state}}}\end{array}$$

Total federal per capita food waste generation was calculated based on a population-weighted average to capture total food waste generation across all states while accounting for the fact that larger states exert more weight than smaller states on the final value:

2 $$\begin{array}{l}{\mathrm{Federal}}\; {\mathrm{per}}\; {\mathrm{capita}}\; {\mathrm{wasted}}\; {\mathrm{food}}\; {\mathrm{generation}}\\=\frac{\mathop{\sum }\nolimits_{i=1}^{50}{\left({\mathrm{Total}}\; {\mathrm{food}}\; {\mathrm{waste}}\; {\mathrm{generation}}\; {\mathrm{of}}\; {\mathrm{state}}\;({\mathrm{Mt}})\right)}_{i}\times907.185\,({\mathrm{kg}}\;{\mathrm{Mt}}^{-1})}{\mathop{\sum }\nolimits_{i=1}^{50}{({\mathrm{Population}}\; {\mathrm{size}}\; {\mathrm{of}}\; {\mathrm{state}})}_{i}}\end{array}$$

### Solution-to-policy allocation

Solutions documented in ReFED’s Solutions Database (https://insights-engine.refed.org/solution-database) were assigned to the most relevant policy intervention type listed in ReFED’s Policy Finder (date labelling, liability protection, tax incentives, animal feed and organic waste bans/waste recycling laws) and corresponding broad policy category (Extended Data Table 4). Two ‘solutions’ scenarios were modelled: a *baseline* scenario, which considered a set of solutions deemed most relevant to each policy category and an *alternative* scenario, which included additional solutions that may indirectly benefit from a given policy (for example, educational campaigns). To avoid double counting, the diversion potential of any given solution was accounted for only once by assigning it to a single policy category, with some exceptions. For example, because liability protection and tax incentives both aim to increase food donations rates for surplus food, a basic 50/50 allocation method was applied. In this case, 50% of the diversion potential of a relevant solution was allocated to liability protection and tax incentives (each). The same approach was applied to the consumer education campaigns to reflect the fact that such campaigns may concurrently target food preparation, storage and use (contributing towards wasted food prevention) and food waste sorting and recycling behaviour (contributing towards food waste recycling).

### Applicable food waste diversion potential quantification

To estimate the applicable food waste diversion potential (in Mt) across states, we utilized estimates from ReFED’s Solutions Database. The database quantifies the food waste diversion potential of feasible solutions across the food supply chain. The solutions modelled have all been demonstrated as actionable and impactful strategies to address food waste and were informed by publicly and privately available sources and expert consultations.

The following key indicators were used to estimate the applicable food waste diversion potential for each solution:

1. Applicability rate (by sector, cause and food type): a quantitative unit (typically represented as a 0 or 1) that identifies, within a sector, the proportion of food waste attributed to a given cause a solution is relevant for (depending on food type), to estimate addressable waste streams; and
2. Diversion rate (by sector and food type): the percentage of any addressable waste stream a solution can prevent or redirect toward the preferable management pathway, based on the applicable streams of food waste as determined above.

In most circumstances, a variety of solutions might be employed to prevent the same amount of food from going to waste. To avoid double counting, ReFED’s model employs a ‘waterfall’ approach, whereby solutions are implemented in a ranked order so that food waste addressed by a given solution is subtracted from the total amount that subsequent solutions can address. Solutions are ordered considering their position within the EPA’s Food Recovery Hierarchy, their logical implementation order and their net financial benefit.

### Policy scoring and likely food waste diversion potential quantification

Next, we estimated the likely food waste diversion potential range (in Mt) by applying a policy scoring matrix, informed by ReFED’s Policy Finder (https://policyfinder.refed.org/), to the applicable food waste diversion potential. The Policy Finder assigns a qualitative impact classification (*negative*, *non-existent*, *weak*, *moderate* and *strong*) for each of the five policy intervention types. A conversion matrix was then applied to convert qualitative scores into quantitative factors (on a 0–1 range) to capture the level of confidence in the potential of a given policy to reduce food waste (Extended Data Table 5). To account for intrinsic variability associated with policy effectiveness, each score was attributed a pair of high and low factors that provided the lower and upper limits of the subsequent diversion potential ranges. Without empirical evidence on quantitative policy assessment, factors were approximated based on expert consultations. Estimates were calculated across a range instead of a single value to reflect the uncertainty of implementation effectiveness for any given policy.

Individual likely food waste diversion potential ranges (by policy category) were summed to obtain a total diversion range for each state. Likely per capita food waste diversion potential values were calculated for each factor *f* (high and low) and scenario *s* (baseline and alternative) based on the following equation:

3 $$\begin{array}{l}{\mathrm{State}}\; {\mathrm{likely}}\; {\mathrm{per}}\; {\mathrm{capita}}\; {\mathrm{food}}\; {\mathrm{waste}}\; {\mathrm{diversion}}_{f,s}\\=\frac{{\mathrm{Applicable}}\; {\mathrm{food}}\; {\mathrm{waste}}\; {\mathrm{diversion}}\; {\mathrm{of}}\; {\mathrm{state}}_{s}\,\left({\mathrm{Mt}}\right)\times{f}\times907.185\,({\mathrm{kg}}\;{\mathrm{Mt}}^{-1})}{{\mathrm{Population}}\; {\mathrm{size}}\; {\mathrm{of}}\; {\mathrm{state}}}\end{array}$$

Similarly to total federal per capita food waste generation (equation (2)), a population-weighted approach was used to scale up the likely per capita food waste diversion potion for each factor *f* and scenario *s* across the 50 states *i*:

4 $$\begin{array}{l}{\mathrm{Federal}}\; {\mathrm{likely}}\; {\mathrm{per}}\; {\mathrm{capita}}\; {\mathrm{food}}\; {\mathrm{waste}}\; {\mathrm{diversion}}_{f,s}\\=\frac{\mathop{\sum }\nolimits_{i=1}^{50}{\left({\mathrm{Applicable}}\; {\mathrm{food}}\; {\mathrm{waste}}\; {\mathrm{diversion}}\; {\mathrm{of}}\; {\mathrm{state}}_{s}\left({\mathrm{Mt}}\right)\times{f}\;\right)}_{i}\times907.185\,({\mathrm{kg}}\;{\mathrm{Mt}}^{-1})}{\mathop{\sum }\nolimits_{i=1}^{50}{({\mathrm{Population}}\; {\mathrm{size}}\; {\mathrm{of}}\; {\mathrm{state}})}_{i}}\end{array}$$

### Revised likely food waste diversion potential

A sensitivity analysis was performed to reflect the distinction between the EPA’s original (2016) and revised (2021) food waste definition, referred to as EPA-2016 and EPA-2021, respectively. This distinction enabled the assessment of the relative contribution of recycling solutions compared to prevention and rescue pathways, because, upon revision, the EPA no longer included recycling solutions (that is, solutions listed under the ‘Organic Waste Bans & Recycling Laws’ policy intervention) as viable contributions towards food waste diversion. Thus, the relationship between the EPA-2016 and EPA-2021 diversion potential (in kg per capita) at the state and federal levels were calculated as follows:

5 $$\begin{array}{l}{\mathrm{EPA}} \sim 2021\;{\mathrm{state}}\; {\mathrm{per}}\; {\mathrm{capita}}\; {\mathrm{diversion}}\; {\mathrm{potential}}_{f,s}\\\qquad\;\,={\mathrm{EPA}} \sim 2016\;{\mathrm{state}}\; {\mathrm{diversion}}_{f,s}\left[{\mathrm{total}}\right]\\\qquad\;\,-{\mathrm{EPA}} \sim 2016\;{\mathrm{state}}\; {\mathrm{diversion}}_{f,s}\;[\mathrm{recycling}]\end{array}$$

6 $$\begin{array}{l}{\mathrm{EPA}} \sim 2021\;{\mathrm{federal}}\; {\mathrm{per}}\; {\mathrm{capita}}\; {\mathrm{diversion}}\; {\mathrm{potential}}_{f,s}\\=\frac{\mathop{\sum }\nolimits_{i=1}^{50}{\left({\mathrm{EPA}} \sim 2021\;{\mathrm{food}}\; {\mathrm{waste}}\; {\mathrm{diversion}}\; {\mathrm{of}}\; {\mathrm{state}}_{f,s}\left(\mathrm{Mt}\right)\right)}_{i}\times907.185\,({\mathrm{kg}}\;{\mathrm{Mt}}^{-1})}{\mathop{\sum }\nolimits_{i=1}^{50}({\mathrm{Population}}\; {\mathrm{size}}\; {\mathrm{of}}\; {\mathrm{state}})_{i}}\end{array}$$

### Projected food waste generation levels

In the final step, projected food waste generation levels (in kg per capita) for the EPA-2021 scenario were estimated at the state and federal level based on the following equations:

7 $$\begin{array}{l}{\mathrm{Projected}}\; {\mathrm{state}}\; {\mathrm{per}}\; {\mathrm{capita}}\; {\mathrm{food}}\; {\mathrm{waste}}\; {\mathrm{generation}}_{f,s}=\\{\mathrm{Current}}\; {\mathrm{state}}\; {\mathrm{per}}\; {\mathrm{capita}}\; {\mathrm{food}}\; {\mathrm{waste}}\; {\mathrm{generation}}\\-{{\mathrm{EPA}} \sim 2021\;{\mathrm{state}}\; {\mathrm{diversion}}\; {\mathrm{potential}}}_{f,s}\end{array}$$

8 $$\begin{array}{l}{\mathrm{Projected}}\; {\mathrm{federal}}\; {\mathrm{per}}\; {\mathrm{capita}}\; {\mathrm{food}}\; {\mathrm{waste}}\; {\mathrm{generation}}_{f,s}=\\{\mathrm{Current}}\; {\mathrm{federal}}\; {\mathrm{per}}\; {\mathrm{capita}}\; {\mathrm{food}}\; {\mathrm{waste}}\; {\mathrm{generation}}\\-{{\mathrm{EPA}} \sim 2021\;{\mathrm{federal}}\; {\mathrm{diversion}}\; {\mathrm{potential}}}_{f,s}\end{array}$$

### Reporting summary

Further information on research design is available in the Nature Portfolio Reporting Summary linked to this article.

## Supplementary information

Reporting Summary

## Source data

Source Data Fig. 1 Statistical source data at the federal level.

Source Data Figs. 2–5 Statistical source data at the state level (used for Figs. 2–5).

## Figures and Tables

**Extended Data Table 1:**
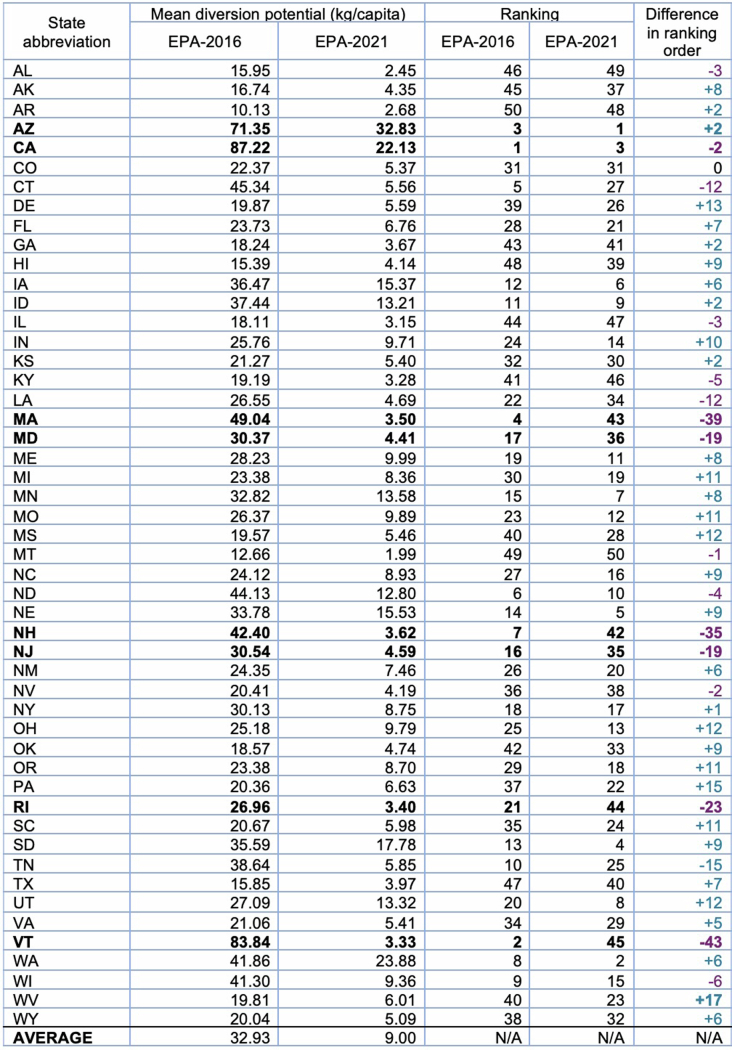
Mean and ranking for likely food waste diversion potential (2022) when including (EPA-2016 scenario) or excluding recycling (EPA-2021 scenario)

The top three states and/or states with a difference in ranking order > |20| are highlighted in bold. Yellow: rise in rank; blue: fall in rank.

**Extended Data Table 2:**
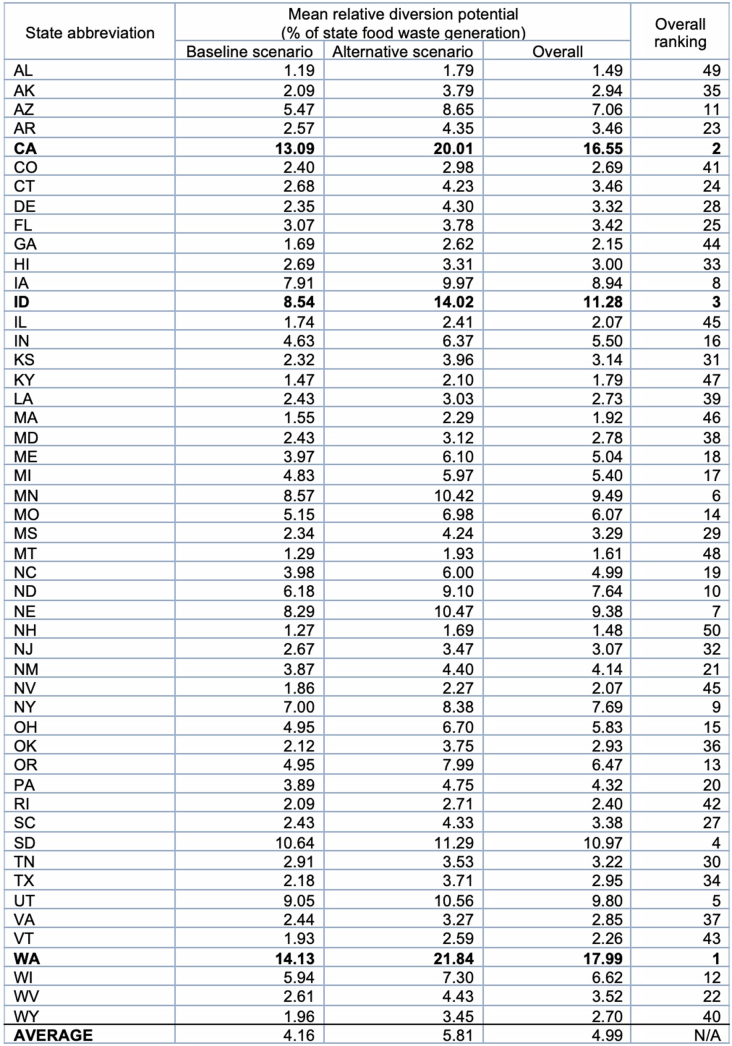
Mean likely food waste diversion potential relative to current state food waste generation levels (2022) under the EPA-2021 scenario

The top (overall) three states are highlighted in bold.

**Extended Data Table 3:**
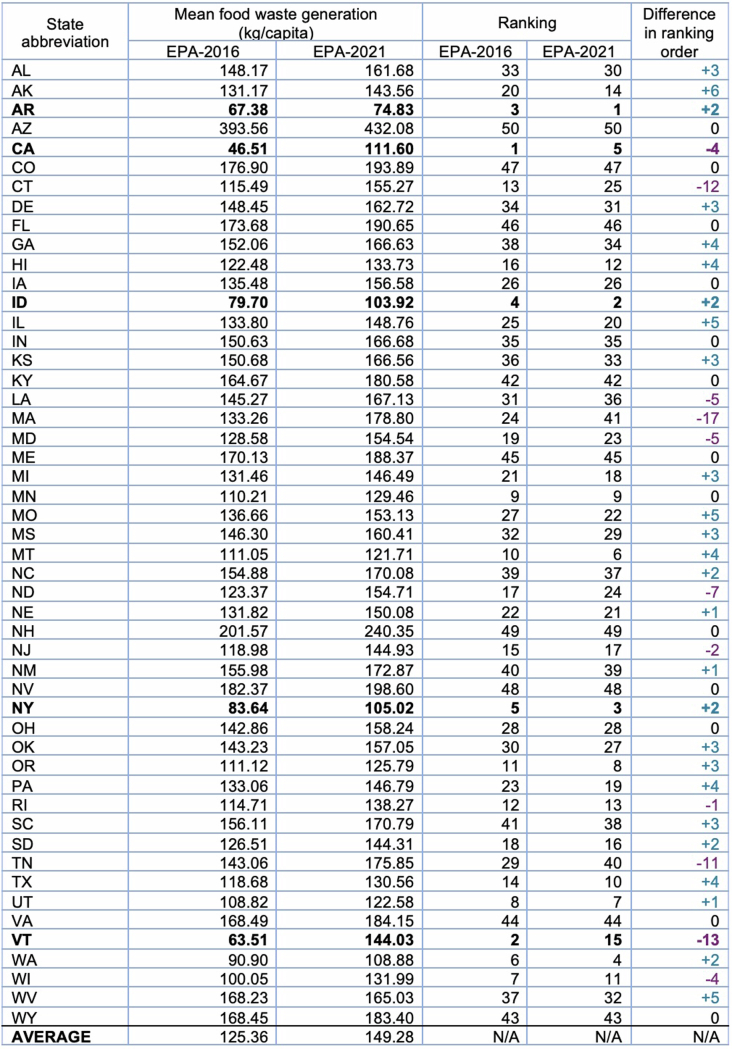
Mean and ranking for likely food waste generation (2022) when including (EPA-2016 scenario) or excluding recycling (EPA-2021 scenario)

The top three states in either scenario are highlighted in bold. Yellow: rise in rank; blue: fall in rank.

**Extended Data Table 4:**
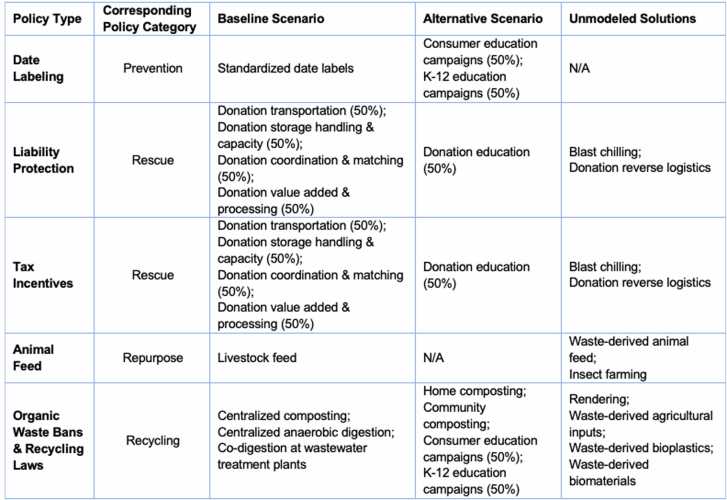
Policy solutions allocation matrix to determine applicable waste food diversion potential

The naming of policy intervention types and solutions followed ReFED’s terminology. Scenarios for each policy category include all solutions from their respective baseline scenario and the additional solutions listed. Most solutions were assigned to a single policy category, with some exceptions, where a 50/50 allocation method was applied (50%) for any given scenario. Unmodeled solutions represent relevant solutions for which no data currently exists but may be added to the baseline scenario in the future.

**Extended Data Table 5:**
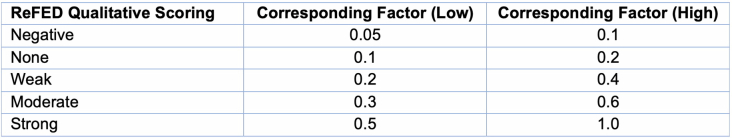
Policy scoring conversion matrix

Each policy was assigned a low and high conversion factor based on its corresponding score on ReFED’s ordered scale (negative, non-existent, weak, moderate, and strong). The factors represent the lower and upper limit of confidence in the effectiveness of a given policy intervention type.

## Data Availability

Primary and secondary sources and data supporting the findings of this study were all publicly available at the time of submission and were downloaded as CSV files from the following websites: ReFED’s Insights Engine Food Waste Monitor (https://insights-engine.refed.org/food-waste-monitor) and Solutions Database (https://insights-engine.refed.org/solution-database), ReFED’s Policy Finder (https://policyfinder.refed.org) and the US Census Bureau (https://www.census.gov). All input and output data files used or generated during this study are included in this published article and via GitHub at https://github.com/s-kakad/wasted_policy. Source data are provided with this paper.

## Code availability

The authors confirm that the code used for data import, processing and analysis during this study are included in this published article via GitHub as R scripts at https://github.com/s-kakad/wasted_policy. The code pipeline was developed and written in R (v4.4.1) via RStudio (v2024.04.2+764).
