# Supplementary material for: State-level policies alone are insufficient to meet the federal food waste reduction goal in the United States
Source: Nat Food. 2025 Jan 9;6(2):196–204. doi: 10.1038/s43016-024-01092-w (PMC11850282; doi:10.1038/s43016-024-01092-w)
Supplement: Supplementary file 1 — Reporting Summary [file 43016_2024_1092_MOESM1_ESM.pdf]

nature portfolio

Corresponding author(s): Sarah Kakadellis

Last updated by author(s): Nov 1, 2024

Reporting Summary

Nature Portfolio wishes to improve the reproducibility of the work that we publish. This form provides structure for consistency and transparency in reporting. For further information on Nature Portfolio policies, see our Editorial Policies and the Editorial Policy Checklist.

Statistics

For all statistical analyses, confirm that the following items are present in the figure legend, table legend, main text, or Methods section.

n/a	Confirmed	
☐	☒	The exact sample size (*n*) for each experimental group/condition, given as a discrete number and unit of measurement
☐	☒	A statement on whether measurements were taken from distinct samples or whether the same sample was measured repeatedly
☐	☒	The statistical test(s) used AND whether they are one- or two-sided<br>*Only common tests should be described solely by name; describe more complex techniques in the Methods section.*
☒	☐	A description of all covariates tested
☐	☒	A description of any assumptions or corrections, such as tests of normality and adjustment for multiple comparisons
☐	☒	A full description of the statistical parameters including central tendency (e.g. means) or other basic estimates (e.g. regression coefficient) AND variation (e.g. standard deviation) or associated estimates of uncertainty (e.g. confidence intervals)
☐	☒	For null hypothesis testing, the test statistic (e.g. *F*, *t*, *r*) with confidence intervals, effect sizes, degrees of freedom and *P* value noted<br>*Give P values as exact values whenever suitable.*
☒	☐	For Bayesian analysis, information on the choice of priors and Markov chain Monte Carlo settings
☒	☐	For hierarchical and complex designs, identification of the appropriate level for tests and full reporting of outcomes
☒	☐	Estimates of effect sizes (e.g. Cohen's *d*, Pearson's *r*), indicating how they were calculated

Our web collection on statistics for biologists contains articles on many of the points above.

Software and code

Policy information about availability of computer code

Data collection	Data was downloaded from publicly available data sources as CSV files from the following website on a Google Chrome web browser: ReFED's Insights Engine (https://insights.refed.org), ReFED’s Policy Finder (https://policyfinder.refed.org), and the US Census Bureau (https://www.census.gov).
Data analysis	All analysis was performed in R (v4.4.1) via RStudio (v2024.04.2+764). The pipeline and data input and output files are available in the GitHub repository: github.com/s-kakad/wasted_policy.

For manuscripts utilizing custom algorithms or software that are central to the research but not yet described in published literature, software must be made available to editors and reviewers. We strongly encourage code deposition in a community repository (e.g. GitHub). See the Nature Portfolio guidelines for submitting code & software for further information.

Data

Policy information about availability of data

All manuscripts must include a data availability statement. This statement should provide the following information, where applicable:

- Accession codes, unique identifiers, or web links for publicly available datasets
- A description of any restrictions on data availability
- For clinical datasets or third party data, please ensure that the statement adheres to our policy

Primary and secondary sources and data supporting the findings of this study were all publicly available at the time of submission and were downloaded as CSV files

from the following websites: ReFED's Insights Engine Food Waste Monitor (https://insights-engine.refed.org/food-waste-monitor) and Solutions Database (https://insights.refed.org/solution-database), ReFED's Policy Finder (https://policyfinder.refed.org), and the US Census Bureau (https://www.census.gov). All input and output data files used or generated during this study are included in this published article through GitHub at the following link: https://github.com/s-kakad/wasted_policy.

Research involving human participants, their data, or biological material

Policy information about studies with human participants or human data. See also policy information about sex, gender (identity/presentation), and sexual orientation and race, ethnicity and racism.

Reporting on sex and gender	Not applicable
Reporting on race, ethnicity, or other socially relevant groupings	Not applicable
Population characteristics	Not applicable
Recruitment	Not applicable
Ethics oversight	Not applicable

Note that full information on the approval of the study protocol must also be provided in the manuscript.

Field-specific reporting

Please select the one below that is the best fit for your research. If you are not sure, read the appropriate sections before making your selection.

☐ Life sciences ☒ Behavioural & social sciences ☐ Ecological, evolutionary & environmental sciences

For a reference copy of the document with all sections, see nature.com/documents/nr-reporting-summary-flat.pdf

Behavioural & social sciences study design

All studies must disclose on these points even when the disclosure is negative.

Study description	This study is a quantitative policy impact analysis that aimed to quantify ranges of food waste diversion and predict future food waste generation levels across the 50 US states, based on existing food-related policies at the state and federal level.
Research sample	The research sample included the 50 US states for the year 2022 representative of the US population and food waste generation within those states. This is based on the data available by the sources used (ReFED's Insights Engine and Policy Finder), which did not have any food waste generation data for the D.C. area, Puerto Rico, and other US territories. The Insights Engine a centralized repository based on data from over 80 public and proprietary databases and provides estimates of food loss, food waste, and food surplus (Food Waste Monitor) and associated amounts of food waste diversion available through a range of strategies (Solutions Database). The Policy Finder assigns a qualitative policy score to food waste reduction policy across five broad categories. Demographic data extracted from the US Census Bureau included state population for the year 2022.
Sampling strategy	All data available for the year 2022 and for the 50 US states were used, within the pre-defined system boundaries (see data exclusions).
Data collection	Data was collected as .csv files from publicly available databases (ReFED and the US Census Bureau) on the corresponding author's work laptop. No other equipment was used. While the researcher carrying out the analysis was not to the study hypothesis, our research was exploratory in nature, which limited the researcher's subjectivity.
Timing	Data collection took place between October 3, 2023 and December 14, 2023. The final datasets used in this study were downloaded on December 14, 2023.
Data exclusions	Food loss and waste data from the farm and manufacturing sectors were omitted when quantifying total food waste generation to align with the US Environmental Protection Agency (EPA)'s methodology, as stated in the manuscript. The contributions of food waste reduction solutions other than the 9 and 14 solutions defined in the baseline and alternative scenarios were also omitted, based on pre-defined policy categories and their corresponding solutions. Please note, both data exclusions took place prior to any formal analysis and interpretation.
Non-participation	This study did not include any participant.
Randomization	Randomization was not relevant to the study design, since the study addressed individual policies for a given state, across the 50 states. Where an allocation method was necessary (e.g. two policies were equally relevant to a given food diversion solution), then a a 50-50% allocation method was applied to avoid introducing bias in the absence of additional evidence.

Reporting for specific materials, systems and methods

We require information from authors about some types of materials, experimental systems and methods used in many studies. Here, indicate whether each material, system or method listed is relevant to your study. If you are not sure if a list item applies to your research, read the appropriate section before selecting a response.

Materials & experimental systems

n/a	Involved in the study
☒	☐ Antibodies
☒	☐ Eukaryotic cell lines
☒	☐ Palaeontology and archaeology
☒	☐ Animals and other organisms
☒	☐ Clinical data
☒	☐ Dual use research of concern
☒	☐ Plants

Methods

n/a	Involved in the study
☒	☐ ChIP-seq
☒	☐ Flow cytometry
☒	☐ MRI-based neuroimaging

Plants

Seed stocks

Issue with the PDF - no plant material was used in this study.

Novel plant genotypes

See above.

Authentication

See above.
